# Supplementary material for: Native Whey Induces Similar Adaptation to Strength Training as Milk, despite Higher Levels of Leucine, in Elderly Individuals
Source: Nutrients. 2019 Sep 4;11(9):2094. doi: 10.3390/nu11092094 (PMC6770720; doi:10.3390/nu11092094)
Supplement: Supplementary file 1 [file nutrients-11-02094-s001.pdf]

Supplementary table 1 Antibodies

Antibody	Dilution	Amount of protein loaded on gel (µg)	Cat. no	Manufacturer
P70S6K[1]	1:1000	30	2708	Cell signaling
phospho-P70S6K Thr^{389}	1:1000	30	9234	Cell signaling
eEF-2[2]	1:5000	30	2332	Cell signaling
phospho-eEF-2^{Thr56}	1:5000	30	2331	Cell signaling
4E-BP1[3]	1:1000	60	9452	Cell signaling
phospho-4EBP-1$^{Thr37/46}$	1:1000	60	9455	Cell signaling
Secondary anti-rabbit	1:3000		7074	Cell signaling

[1] *Ribosomal protein S6 kinase*

[2] *Eukaryotic elongation factor 2*

[3] *Eukaryotic translation initiation factor 4E-binding protein 1*
